# Stochastic Forecasting of Labor Supply and Population: An Integrated Model

**DOI:** 10.1007/s11113-017-9451-3

**Published:** 2017-11-29

**Authors:** Johann Fuchs, Doris Söhnlein, Brigitte Weber, Enzo Weber

**Affiliations:** 1 Research Department Forecasts and Macroeconomic Analyses, Institute for Employment Research, Regensburger Str. 104, 90478 Nuremberg, Germany; 2 Faculty of Business, Economics and Management Information Systems, University of Regensburg, 93053 Regensburg, Germany

**Keywords:** Stochastic population forecast, Principal components, Demography, Labor force participation, Labor supply

## Abstract

This paper presents a stochastic model to forecast the German population and labor supply until 2060. Within a cohort-component approach, our population forecast applies principal components analysis to birth, mortality, emigration, and immigration rates, which allows for the reduction of dimensionality and accounts for correlation of the rates. Labor force participation rates are estimated by means of an econometric time series approach. All time series are forecast by stochastic simulation using the bootstrap method. As our model also distinguishes between German and foreign nationals, different developments in fertility, migration, and labor participation could be predicted. The results show that even rising birth rates and high levels of immigration cannot break the basic demographic trend in the long run. An important finding from an endogenous modeling of emigration rates is that high net migration in the long run will be difficult to achieve. Our stochastic perspective suggests therefore a high probability of substantially decreasing the labor supply in Germany.

## Introduction

Europe’s population is expected to undergo a dramatic change due to low fertility on the one hand and high migration on the other. Eurostat projects a declining total as well as working-age population for many EU-28 countries in the long run (Eurostat 2015). The Ageing Report of the European Commission states a “dramatic change in the age structure in the EU” (European Commission 2015, p. 1), where the old-age dependency ratio almost doubles between 2013 and 2060.

These developments may pose long-run challenges, for example, to the social security system. The labor market plays an important intermediate role, as the demographic burden depends on the workforce. Despite high unemployment in several European countries, concerns have been voiced that a lack of well-trained workers will develop for demographic reasons. This raises the questions of how the population develops and how many people will be available to the labor market. To answer these questions, we need to analyze and forecast the population of working age and the labor force participation rates (LFPRs). The present paper delivers a forecast of these two variables for Germany. Being one of the “lowest fertility” countries worldwide, besides Japan, Poland, or Spain, Germany has experienced high net immigration in recent years. With respect to labor participation, it ranks among the top countries in Europe. This constellation, thereby, makes it interesting to research Germany’s prospects.

In addition to the size of the population, its structure is also of interest, particularly with respect to different LFPRs in different demographic groups. In Germany, the LFPRs among young people and older workers, women and particularly female foreign nationals, are relatively low compared with middle-aged men. For this reason, our forecast is disaggregated by gender and age. Furthermore, we separate German and foreign nationals, as they differ in several demographic and labor participation characteristics. A preferable deeper disaggregation by countries of origin, similar to the approach of the US population division, which differentiates by race and ethnicity (United States Census Bureau 2014), still fails for data reasons, but might be feasible in future.

We apply the cohort-component method to predict the population and link this forecast with the participation rates. To reduce dimensionality and to account for the high correlation between the variables, we take advantage of applying principal components analysis (PCA) to the group-specific series. Principal components (PC) have been proven to be appropriate for estimating demographic components, particularly mortality, being popularized by Lee and Carter (1992). Using the PC approach for the rates of the population change, i.e., birth, mortality, immigration, and emigration, allows for a much more efficient modeling of their age pattern (Booth and Tickle 2008).

Relying on the estimated PC means that only the first few PC, which explain most of the variance in the data, have to be forecast. These predicted PC will be used in the regression equation to forecast the variables of interest. PCA simplifies matters in the case of many, often highly correlated time series, e.g., age-specific mortality rates and birth rates.

In order to estimate and project all the influential variables, we construct a simulation model that describes all demographic components as endogenous variables. Births, deaths, migration, and naturalizations as well as participation rates are analyzed on the basis of time series and modeled stochastically.

Stochastic approaches have become widely used in the field of long-term population forecasting (e.g., Sanderson et al. 2004), partly in response to criticism with respect to the deterministic methodology that typically provides scenarios based on different sets of assumptions (Lee and Tuljapurkar 1994). One of the main disadvantages is that the deterministic approach does not attach any probabilities to the scenarios, insofar as the user cannot compare the uncertainty of different projection scenarios. Even more, the way the assumptions are bundled into scenarios results in a somewhat inconsistent probabilistic interpretation (for a comprehensive discussion, see National Research Council 2000, 190f.). The Ageing Report of the EU, cited above, depicts a further problem: the projected developments are based on a set of assumptions that are not uniformly accepted by all member states of the EU (European Commission. Directorate General for Economic and Financial Affairs 2015, Chapter 1). In contrast, in case of a probabilistic approach, such predefinitions can be discussed upon a clear statistical basis, including model structure and selection criteria.

The stochastic approach systematically exploits the information contained in data based on objective modeling criteria. It simultaneously considers the complex interactions between the multiple components. The modeling framework is inherently open for modeling additional linkages that can be based on theoretical considerations. Moreover, unlike deterministic models, our approach makes it possible to specify the related confidence intervals in addition to point estimates.

There are only few studies that provide a stochastic forecast of LFPR, mostly based on time series modeling (e.g., Frees 2003). To our knowledge, a combined stochastic forecast of population and labor force has not been published elsewhere.^1^ Our model integrates a stochastic forecast of both and, as a result, a fully stochastic prediction of the labor force can be offered.

The next section outlines the basic model structure. The data material used is detailed thereafter. Section “Estimates and Forecast” describes the methodology and statistical details. In Section “Results,” our forecasts are presented and discussed. The last section summarizes and concludes.

## Data and Methods

### Methodological Framework

The probabilistic population forecast relies on the widely used cohort-component method (e.g., Rowland 2003), i.e., it starts from an initial population, which is disaggregated by age, sex and German nationals/non-nationals. As far as the population components are concerned, birth and mortality rates, migration, and naturalizations are modeled by PC. These PC are projected using time-series methods to yield the population forecast (for an overview, see Alders et al. 2007). The inputs for the model are the size and structure of the population in the above disaggregation in Germany for the base year of 2013. The intermediate result is the population for the subsequent year.

Our model distinguishes between immigration and emigration. These are also modeled differently: immigration is initially estimated as an aggregate, distinguishing between German and foreign nationals, and then distributed by gender and individual year of age using projected ratios (top-down). In contrast, emigration is estimated as a proportion depending on the respective population group. For this reason, emigration increases as the population grows. Our approach ensures that emigration will never exceed the population in any age group.

As for the population, the LFPRs are disaggregated by age, sex, and nationality and estimated by means of an econometric time series approach. To calculate the labor force, the participation rates are combined with the respective population.

With these estimates, probability distributions for fertility, mortality, migration, and LFPR were derived by bootstrapping, based on 5000 random draws. The number of simulations was restricted for computational reasons; however, it is quite high compared with other studies (e.g., Alders et al. 2007; Sanderson et al. 2004). During the design and modeling phase, we merely run 1000 simulations. Comparing these intermediate stage results to the final results showed a high stability.

### Data

The population data are obtained from the Federal Statistical Office (FSO) until 2013. The data are broken down into German and foreign nationals, with each group then differentiated further by gender and individual year of age (aged 90 or older combined to one group). The 2013 population structured according to these attributes is the initial population for this model.

In May 2011, a register-based census was conducted in Germany (see FSO 2013). This “2011 census” resulted in a considerable break in the official population count. The population in Germany was corrected downward by approx. 1.5 million, or 1.8%, to 80.2 million—compared to the number of inhabitants from the current population estimate. The correction was particularly drastic with regard to foreign nationals: the new number of foreign nationals as of the census deadline was by 1.1 million (14.9%) lower than before. Since the population figures are used in many other population statistics (e.g., mortality rates, birth rates), these changes result in breaks in the respective time series.

The FSO provides birth rates of single-year age groups (15–49 years), broken down by German and foreign mothers, up to 2013 (starting with 1991). The 2011 census implicates a significant jump in the age-specific birth rates of female foreign nationals. Since the number of births was not affected by the revision but the census yielded a smaller population, the age-specific birth rates changed (FSO 2015). Therefore, an increase in the total fertility rate (TFR) for female foreign nationals, from 1.6 (in 2010) to approx. 1.8 children per woman in 2011, is partly a result of the census population revision.

We use age-specific mortality rates from the life tables from 1957/58 to 2010/12 for Germany (territory of the former Federal Republic of Germany until 1990). These rates do not differentiate between German and non-Germans, because appropriate data from the FSO, which covers a longer period of time, are not available. The reliability of mortality rates from other sources unfortunately appears to be highly questionable (see Zur Nieden and Sommer 2016). However, this should not result in a noticeable distortion in the projected working-age population, as the mortality rates for the relevant age interval are very low. Even a much lower (or higher) mortality would have only a limited impact (see Fuchs and Söhnlein 2006).

Since our model distinguishes between nationals and non-nationals, it is necessary to take into account the number of naturalizations. Age- and sex-specific naturalization figures from FSO were available for 1998–2013.

The migration data segregate immigration and emigration of German and foreign nationals, by gender and by individual year of age from zero to 90 years. The time series cover the period from 1991 to 2014. An exception is the immigration of foreigners, where preliminary values are provided for 2015.

The LFPRs are taken from the labor force survey. For Germany, as a whole, these data were available from 1991 to 2013. Our approach of the labor force participation incorporates an estimate for the hidden unemployed (the hidden labor force) (see Agbola 2005; Armstrong 1999). In the next section, the estimation of these potential LFPRs is set out in detail.

### Estimations and Forecasting

#### Estimations of Principal Components

For each demographic component and for each sex, we apply PCAs to account for the correlation between the demographic rates and to reduce dimensionality (Lee and Carter 1992). A singular-value decomposition generates from the k time series a set of k PC which are orthogonal to each other. By construction, the first PC accounts for the largest part of the variance because they are arranged in a descending order. Taking the first j PC (with j < k) reduces dimensionality of the model. These PC are projected and used to forecast the k different time series for fertility, mortality, naturalization, and migration.

In our model, the time series are mostly linear combinations of more than one PC. For example, the age-specific fertility rates for German women $cfr_{t,i}^{G}$ for age groups *i* = 15,…, 49 are modeled as

$$ln\left( {cfr_{\text{t,i}}^{\text{G}} } \right) = c_{1} + \mathop \sum \limits_{k = 1}^{n} c_{k + 1 } pc_{k,t} + \varepsilon_{t}$$

with $pc_{\text{k,t}} , k = 1, \ldots ,n$, being the first n principal components for $ln\left( {cfr_{\text{t,i}}^{\text{G}} } \right)$ and $\varepsilon_{t}$ the error term. Taking the logarithm ensures that the fertility rates remain positive.

Various extensions and refinements of the Lee-Carter model can be found in the literature (e.g., Hansen and Pflaumer 2011; Lee and Miller 2001; Lee and Tuljapurkar 1994). In addition to various parametric models, Booth and Tickle (2008) describe several extensions of the Lee-Carter approach, for example with higher order terms, i.e., using more than one PC. This method results in a better model fit (Renshaw and Haberman 2003). We therefore apply this extension to our model.

In the case of the age-specific mortality data, which are strongly correlated, it is plausible to limit the number to one PC, as the first PC already explains most of the variance. Table 1 shows an example of the PC of female mortality rates in Germany. The first PC accounts for almost 98% of the variance.

**Table 1:** Principal components analysis for the survival probability of women

PC No.	Eigenvalues	Cumulative	Proportion	Cum. proportion
1	88.1127	88.1127	0.9790	0.9790
2	0.5816	88.6943	0.0065	0.9855
3	0.4552	89.1494	0.0051	0.9905
4	0.1949	89.3443	0.0022	0.9927
5	0.1028	89.4472	0.0011	0.9939
6	0.0915	89.5386	0.0010	0.9949
7	0.0772	89.6159	0.0009	0.9957
8	0.0444	89.6603	0.0005	0.9962
9	0.0365	89.6967	0.0004	0.9966
10	0.0316	89.7283	0.0004	0.9970
11	0.0292	89.7576	0.0003	0.9973
12	0.0280	89.7855	0.0003	0.9976

Log (Survival probability) = log (1 – death rate)

The mortality data do not distinguish between German nationals and non-Germans

Sample: 1959–2012 (54 observations)

Source: Own calculations; data from Federal Statistical Office, Germany

All other age-specific time series (fertility, naturalizations, immigration, and emigration, see Appendix Tables 9, 10, 11, as an example for German women), however, are less collinear, which raises the question of the appropriate number of PC (e.g., Peres-Neto et al. 2005). For practical purposes, we favor the Kaiser-Guttmann criterion: only eigenvectors with an eigenvalue ≥ 1 are used. In most cases, this criterion yields a generally satisfactory explanation of the variance using an acceptable number of PC (variance coverage).

As is evident from Table 2, one or two PC will suffice for the mortality series; for fertility and naturalizations, there are a few more PC, but projecting the migration time series requires significantly more PC to explain more than 90% of the variance.

**Table 2:** Summary of the principal components analysis. *Source* Own calculations; data from Federal Statistical Office, Germany

Group	No			Variance explained (%)
	Data points	Age groups	Components	
Mortality (m)	54	90	2	97
Mortality (f)	54	90	1	98
Fertility (G)	23	35	3	96
Fertility (F)	23	35	3	92
Naturalizations (m)	16	71	4	97
Naturalizations (f)	16	71	4	98
Immigration (G), (m)	24	91	10	94
Immigration (G), (f)	24	91	7	95
Immigration (F), (m)	24	91	11	94
Immigration (F), (f)	24	91	7	95
Emigration (G), (m)	24	91	8	95
Emigration (G), (f)	24	91	7	93
Emigration (F), (m)	24	91	6	95
Emigration (F), (f)	24	91	6	96

*G* Germans, *F* foreign nationals, *m* male, *f* female

#### Forecasting Principal Components

PC are projected in a similar way to Lee and Carter (1992), using stochastic and deterministic trends. There, the first PC is modeled as a random walk with drift. In a more flexible setup, we represent and forecast the PC using autoregressive (AR) or moving-average processes (MA). Since a unit root can be estimated in the lag structure, this flexible modeling also captures nonstationary cases.

For example, a high autocorrelation in the first lags for the second PC of birth rates of German women $pc_{2,t}^{G}$ indicates suitable modeling using AR-terms. Applying an AR(2) process *x*_t_ yields


The models tested range from AR(1) or MA(1) to ARMA(2,2). Following the correlogram of residuals we selected an AR(2) process, where both the Schwarz criterion and the Hannan-Quinn criterion were minimal (see Table 3 and Appendix Table 12). We preferred these criteria over the Akaike information criterion since they allow more parsimonious specification, which is advantageous in limited samples.

**Table 3:** Equation for the 2nd PC concerning birth rates of German women. *Source* Own calculations; data from Federal Statistical Office, Germany

Dependent variable: PC2_F_G; Method: least squares; Sample (adjusted): 1993–2013; PC2_F_G = c1* PC2_F_G(−1) + c2* PC2_F_G(−2)				
	Coefficient	Std. error	*t*-statistic	Probability
c1	1.232221	0.199706	6.17	0.0000
c2	− 0.382546	0.203331	− 1.88	0.0753

*R* ^2^	0.828	Mean dependent var.	0.122	
Adjusted R^2^	0.819	SD dependent var.	1.968	
SE of regression	0.838	Akaike info criterion	2.575	
Sum squared resid.	13.348	Schwarz criterion	2.675	
Log likelihood	− 25.04	Hannan-Quinn criterion	2.597	
		Durbin-Watson statistic	2.245	

The respective first PC generally show a sharp downward or upward trend. This can yield implausible forecasts. On the one hand, there are natural limits (e.g., a 100% probability of dying); on the other hand, some results may be extremely implausible for theoretical considerations. For example, there is certainly an upper limit for a rise in births over a few years.

Therefore, a logistic functional approach was applied for the respective PC: for a given saturation level SN the right-hand side of the expression *pc*_k,t_ = *y* from above is transformed to

$${\text{pc}}_{1,t}^{F} = \frac{\text{SN}}{{1 + { \exp }\left( {\text{y}} \right)}}$$

So for 0 < *pc*_k,t_ < SN follows:

$$ln\left( {\frac{SN}{{{\text{pc}}_{1,t}^{F} }} - 1} \right) = y$$

A PC can also be negative. The logarithm would then not be defined for the term. A parallel shift of the PC by *d* will correct this. However, this will also transform the saturation level SN into SN′

$$\ln \left( {\frac{{{\text{SN}}{^\prime}}}{{{\text{pc}}_{1,t}^{F} + d}} - 1} \right) = y$$

Like the other coefficients, SN or SN′ and *d* are estimated by maximum likelihood.

For example, the first PC in the birth rates for female foreign nationals ${\text{pc}}_{1,t}^{F}$, which is strongly trending, is estimated using a logistic approach. The estimation for the equation

$$ln\left( {\frac{{SN^{ '} }}{{{\text{pc}}_{1,t}^{F} + d}} - 1} \right) = c_{1} + c_{2} t + \rho_{t}$$

yields the parameters in Table 4, where SN′ = 19.51 and *d* = − 9.25. This equation has no autoregressive term, since it does not yield additional explanatory power.

**Table 4:** Equation for the 1st PC concerning birth rates of female foreign nationals. *Source* Own calculations; data from Federal Statistical Office, Germany

Dependent variable: PC1_F_F; Method: least squares; Sample (adjusted): 1991 2013; PC1_F_F = *SN’*/(1 + EXP(*c1* + c2*t)) + *d*				
	Coefficient	Std. error	*t*-statistic	Probability
SN’	19.50762	1.975521	9.87	0.000
c1	9.854861	1.399583	7.04	0.000
c2	− 0.186835	0.026927	− 6.94	0.000
d	− 9.2532	0.942678	− 9.82	0.000

*R* ^2^	0.994	Mean dependent var.	− 1.54E-16	
Adjusted *R* ^2^	0.993	SD dependent var.	5.122	
SE of regression	0.432	Akaike info criterion	1.315	
Sum squared resid.	3.542	Schwarz criterion	1.512	
Log likelihood	− 11.12	Hannan-Quinn criterion	1.364	
*F*-statistic	1.026	Durbin-Watson statistic	1.003	
Prob (*F*-statistic)	0.000			

The method described above is similar for the various elements, but a different approach is used for immigration and emigration: immigration is initially estimated as an aggregate separately for Germans and foreign nationals, and then broken down by age and gender using the projected rates (top–down). Emigration, on the other hand, is determined using the age-specific shares of the respective population, which then yields the total emigration (bottom–up). Applying the predicted emigration rate to the number of people in a population cell, known from the forecast, allows estimating the emigrants for this cell. As emigration rates are sensitive to age and sex, the bottom-up approach is superior to the top-down one since the structure of the population is already estimated by the forecast and we can use this information for the forecast of the total emigration.

In our model, therefore, the migration balance is the result of the estimated figures for emigration and immigration. A similar procedure as for emigration is applied for naturalizations.

#### Estimating Labor Force Participation

A well-known phenomenon is that labor force participation moves cyclically with respect to the development of the economy. Workers withdraw from a deteriorating labor market (e.g., Dernburg and Strand 1966; Österholm 2010), which is called “discouragement effect”. On the other hand, additional labor force participants may enter the labor market in a recession, for example to ensure their family income (e.g., Prieto-Rodríguez and Rodríguez-Gutiérrez 2000). Obviously, unemployment plays a role with regard to labor force participation rates. The importance of this view can be seen when labor market policy finds itself between demographic scarcity of labor on the one part and labor underutilization on the other (see Baum and Mitchell 2010).

In the following, we use the approach of Agbola (2005) to estimate and predict the LFPR. The equations for the LFPR include an indicator specifying the labor market situation, e.g., the official unemployment rate, a group-specific unemployment rate, or the relation between vacancies and the labor force (vacancy rate). Following the literature, the dependent LFPR was transformed into its logit value (e.g., Frees 2003; Tossi 2011). The logit-transformation ensures that the model never forecasts a participation rate less than 0 or more than 100%.

Due to the breakdown by gender, German and foreign nationals, as well as ten age groups covering the age ranges 15–64, equations were estimated for 40 groups.^2^ For each of the 40 demographic groups, an equation of the following type was estimated:

1 $$\ln \left( {\frac{{\hat{a}_{\text{jt}} }}{{\left( {1 - \hat{a}_{\text{jt}} } \right)}}} \right) = logit\left( {\hat{a}_{\text{jt}} } \right) = b_{0} + b_{1} Z_{\text{t}} + cU_{\text{t}}$$

where $\hat{a}_{\text{jt}}$ is the estimated logit of the statistically measured LFPR, a_jt_. *b*_0_, *b*_1_, and *c* are regression parameters*. U*_t_ represents a regressor serving as the indicator for the labor market situation, e.g., the unemployment rate.* Z*_t_ represents all other regressors, e.g., the part-time employment rate, a trend element or the lagged endogenous variable itself (therefore, *Z* and *b* can be vectors). Both *Z* and *U* can also be represented in lagged form. The index j represents the subpopulation and was omitted on the right-hand side for simplicity.

A deterioration in the labor market situation pushes the LFPR down and also involves lower values for the unemployment indicator U. The expected sign for parameter *c* of the unemployment indicator is therefore clearly defined. In the case of discouragement, the sign of the unemployment rate should be negative, whereas the added worker effect would imply a positive sign. For the vacancy rate, which seems to be important in empirical terms especially for men, the sign is reversed: discouragement involves a positive sign, the added worker effect a negative one. Literature for Germany frequently assumes a larger discouragement effect, although both phenomena can appear simultaneously (Fuchs and Weber 2013).

In case of a boom, the cyclical labor market indicator should take a value that could be considered the “full employment value” (Agbola 2005; Weber 2014). If an equation contains the unemployment rate as employment indicator, the full employment value would be a very low unemployment rate. To determine “full employment values,” we considered information about regional unemployment. Like Armstrong (1999) we took a very low regional unemployment rate as a proxy for the “full employment value”.

Let $U_{\text{t}}^{\text{v}}$ be the “full employment value”. Replacing the actual value of *U*_t_ with $U_{\text{t}}^{\text{v}}$, Eq. (2) will yield the logit of a potential LFPR $\hat{a}_{\text{jt}}^{\text{v}}$, i.e., a participation rate under full-employment conditions:

2 $$logit\left( {\hat{a}_{jt}^{v} } \right) = b_{0} + b_{1} Z_{t} + cU_{t}^{v}$$

The full employment value $U_{\text{t}}^{\text{v}}$ may change over time; hence it may contain the time index *t*. The variables in the vector *Z*_t_ represent the other determining factors for labor force participation. For each demographic cell, we selected variables that were known from the literature to be potentially influential. Table 5 lists the regressors that proved to be significant and were included at least in one of the equations estimated.

**Table 5:** Overview of exogenous variables for the labor force participation rates

Variable	Description	Source
Unemployment	Unemployment rate related to dependent civilian labor force	FEA
	Unemployment rate related to civilian labor force (for females, foreign nationals, young workers under the age of 20, women working part-time)	FEA
Job vacancies	Number of vacancies related to dependent civilian labor force	FEA
	Number of vacancies subject to social security contributions related to dependent civilian labor force	FEA
Wages	Average (median) daily pay of employees subject to social security contributions by gender for Germans and foreign nationals (significant variable: male foreign nationals aged 30–49)	FEA
Education	Student ratio by gender and age (significant: men aged: 20–24, 25–29, women aged 20–24)	FSO
Household	Rate of women working part-time	FSO
	Relation between women age x and children age y (with different values for x and y) (significant: women aged 35–39 to children aged 5–9)	FSO
Retirement	Average retirement age by gender (significant: men aged 60–64)	DRV

*FEA* Federal Employment Agency, Germany, *FSO* Federal Statistical Office, Germany, *DRV* Deutsche Rentenversicherung Bund (German Federal Pension Insurance)

The estimate for German women aged 45–49 is shown as an example in Table 6. The dependent variable is the logit of the LFPR. The equation was estimated by weighted least squares, as the LFPR, just like the logits, are heteroscedastic. The time series of the LFPR cover the period from 1991 to 2013. Significant regressors were the unemployment rate for women, the ratio of women working part-time and a step dummy variable for 2005. The dummy variable represents a fundamental labor market reform that became effective in 2005.

**Table 6:** LFPR equation, German women aged 45–49. *Source* Own calculations; data from the German Labor Force Survey

Dependent variable: Logit of the LFPR German women, aged 45–49	Coefficient	Std. error	Prob.
Unemployment rate for women	− 0.11380	0.003723	0.0065
Rate of women working part-time	0.065462	0.001308	0.0000
Dummy 2005	− 0.718652	0.063438	0.0000
Constant	− 0.153828	0.016360	0.0000
Adjusted *R* ^2^	0.9899		
*F*-statistic	723.58		0.0000
Schwarz criterion	− 3.788		
Breusch-Godfrey serial Correl. LM Test	1.7584		0.42 (n.s)

Dummy 2005: 0 from 1991 to 2004, 1 afterward

Sample: 1991 to 2012

The use of the potential LFPR is equivalent to the approach of the U.S. Bureau of Labor Statistics which predicts long-run participation rates based on the assumption of “… a long-run full-employment economy in which unemployment is frictional and not a consequence of deficient demand.” (Tossi 2011, p. 25). Accordingly, a projection of potential LFPR does not require a forecast of the economic or labor market performance. In addition, a potential LFPR implicitly includes some labor reserves as the hidden unemployed are part of it.

In the equation above, the ratio of women working part-time was extrapolated by a time trend, as the other explaining variables. While this is a widespread practice, future research could experiment with adopting the PC approach to the LFPR. The present approach, however, offers a high flexibility at the modeling stage, as we observe different trends of the demographic groups and specific influencing factors of labor participation.

## Results

Below we present the key results of the stochastic forecasts, each including the average (median) and the confidence interval. The confidence bounds, based on 5000 simulation runs (bootstrap generated), not only show the variability of the residuals, but also include uncertainty in the coefficients. A 66% confidence level was selected for the following graphical representations of our results. The median is preferred over the arithmetic mean as it is more stable with respect to outliers. Note that confidence bands result from the variance of the shocks, but cannot take into account the potential occurrence of major structural breaks in the future.

The forecast horizon is 2060, which allows the comparison of our results with many other long-term forecasts. This forecast horizon shows, in addition, some interesting developments caused by long-run baby boom effects. The growing uncertainty of the forecast is reflected by widening confidence intervals. The latter is a unique feature of the stochastic forecast that shows an important advantage compared to deterministic models.

### Components of the Population Forecast

Life expectancy is expected to rise from 77.7 (current value from the 2010/2012 life table) to 86.1 years for newborn boys and from 82.8 to 89.6 years for newborn girls (Table 7). In other words, our model predicts a reduction in the size of the mortality gap between genders, which corresponds to the development in Germany since the late 1970s (Eisenmenger and Emmerling 2011). According to our estimates, the 66% confidence interval for newborn boys is between 84.5 and 86.4 years in 2060 and therefore relatively small. The survival probabilities can therefore be forecast quite well from the historical data. The confidence interval is similar for newborn girls.

**Table 7:** Life expectancy in Germany, over selected years. *Source* FSO and own calculations

Life expectancy at birth: 2012 base year, afterward median forecast		
	Women	Men
2012	82.80	77.72
2015	83.40	78.31
2020	84.28	79.39
2030	85.83	81.33
2040	87.21	83.08
2050	88.48	84.67
2060	89.61	86.11

The TFR for female foreign nationals is predicted to decline from the current level of 1.8 children per woman in 2013 to 1.77 in 2060. The confidence interval widens in the forecast period from 0.1186 (difference of the TFR) in 2020 to 0.1421 in 2030. Afterward, the difference between the upper and the lower bands only slightly increases from 0.1509 in 2040 up to 0.1580 in 2060. We interpret this narrow interval as a result of the comparable high stability of the age-specific birth rates.

Forecasting fertility of non-German women using PC shows therefore a convergence toward the lower fertility of German women that could be expected from the relevant literature (e.g., for Germany, see Krapf and Wolf 2015; for Europe, see Sobotka 2008). Furthermore, the rise in the number of refugees, mainly young persons, could have boosted the birth rates of German non-nationals since 2011. In the long run, we can expect that the number of refugees will decrease to a “normal” level, so that the extra TFR effect reverts.

**Fig. 1:**
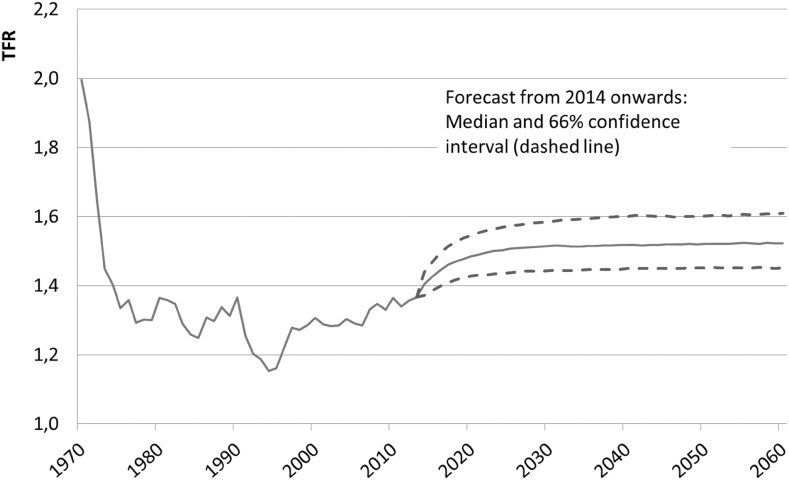
Total fertility rate (TFR) for German women, 1970–2060, median forecast and confidence interval from 2014 onward. *Note* From 1970 to1989 territory of the former Federal Republic of Germany and former East Germany, from 1990 onwards all of Germany. *Source* Federal Statistical Office and own estimations

Among German women the TFR is forecast to increase from 1.37 children in 2013 to 1.48 in 2060 (see Fig. 1). This can be attributed to the age-specific birth rates for women aged 30 and above. The averaged birth rate of the 30–39 year olds is predicted to rise by 20% from 2013 to 2060, while fertility of the 40–49 year olds even surge by 32%. Slightly declining birth rates for women under 30, in contrast, have less impact on the overall development (− 4.1%).

This increase to almost 1.5 births per woman corresponds to what demographers expect for Germany in the long term based on the “tempo effect”. This tempo effect results from the shifts in the age-specific birth rates toward older age groups (i.e., mothers are having children at an increasingly higher age), provided that the delayed births do not ultimately become nonexistent (Luy and Pötzsch 2010). Since—by virtue of nature—this process can ultimately be expected to end, one can expect a rise in the TFR. Insofar, recent studies for the tempo effect support our forecast of the TFR.

Since the reunification in 1990, the average net immigration of non-Germans has been approx. 200,000 persons per year. In the recent years, however, Germany has experienced mass immigration of people from eastern and southern EU countries as well as from countries suffering from armed conflicts (Fuchs et al. 2016). This migration movement reached its peak in 2015 when almost 2 million people came to Germany. Immigration from the EU is expected to settle down in the medium run (Bertoli et al. 2016), and the extraordinary influx of refugees at the early stage of our forecast period has become noticeable weaker. To reflect these exceptional influences, an add-on as a markup on the estimated immigration with a regressive trend was incorporated into the model. It starts with 340,000 persons in the year 2016 and ends in 2025.

Our model predicts a declining number of immigrants in the near future, with the lowest level of 865,000 migrants in 2023. Because immigration follows a positive trend in the long run, it will start to rise again thereafter. Due to our median forecast more than 1.1 million non-Germans will immigrate in 2060.

As the model links emigration to population size, the number of emigrants follows the immigration flows. During the time period 2016–2023, more than 800,000 persons are predicted to leave Germany per year. The considerable immigration will obviously be partly balanced out by rising emigration figures. At the end of the forecast horizon, yearly emigration will be slightly less than 1 million people.

**Fig. 2:**
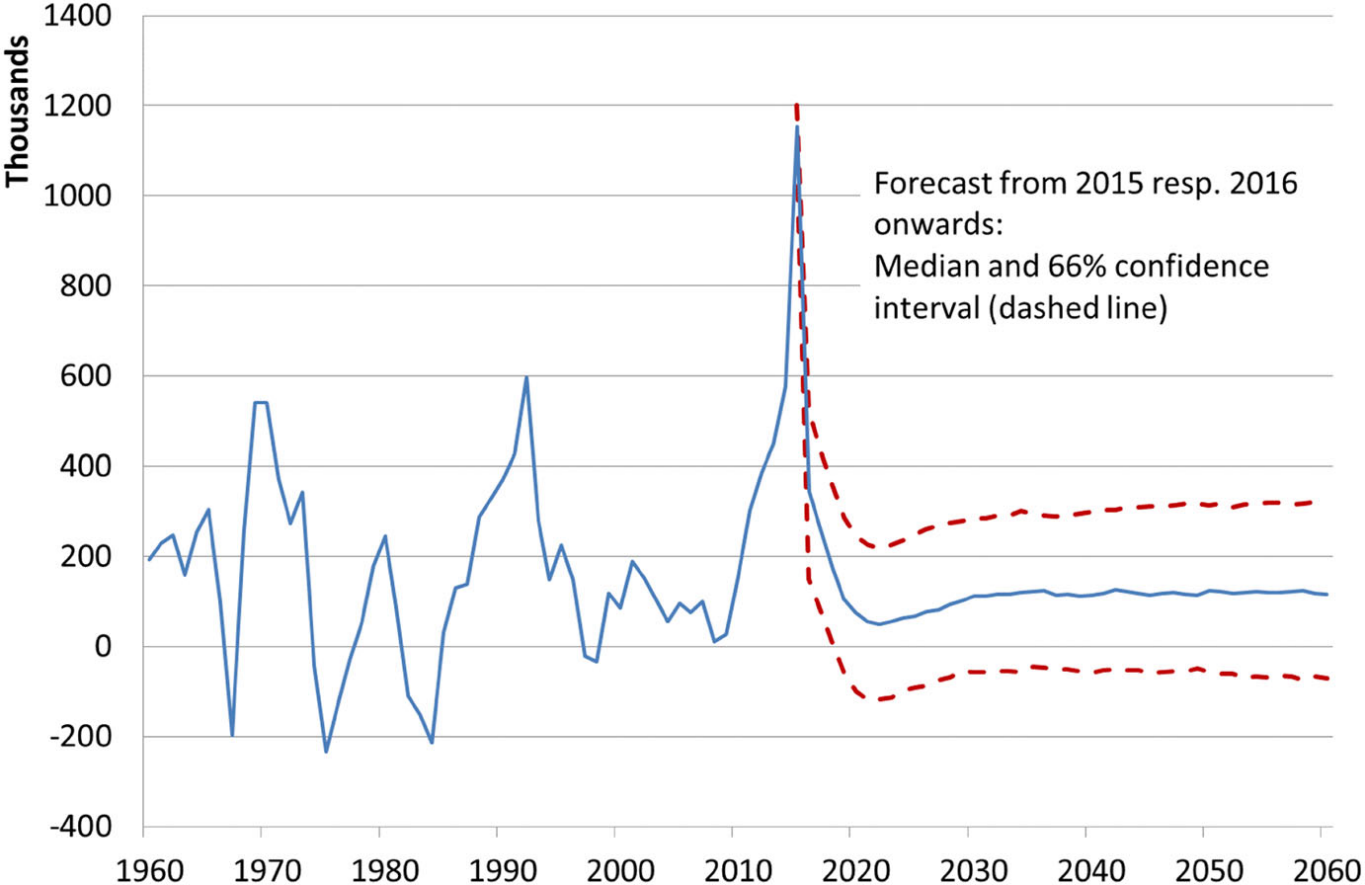
Migration balance for foreign nationals, 1960–2060, median forecast and confidence interval from 2015 onward (emigration) resp. from 2016 onward (immigration). *Note* As for Fig. 1 *Source* Federal Statistical Office and own estimations

Net immigration during the forecast period thus declines from the currently high level (over 1 million in 2015) to about 50,000 migrants in the early 2020th. Afterward, the migration balance increases to 120,000 foreign nationals per year in 2030 and, as Fig. 2 shows, remains almost stable during the rest of the forecast period.

Migration of German nationals was not significant in quantitative terms in recent years. In the last 10 years, the average number of German emigrations exceeded the number of immigrations by 35,000 per year. According to the latest available data, the net migration of German nationals equaled approx. − 26,000 in 2014 (0,035% of the population with German nationality). Our model predicts a net immigration in the long run with a median of + 25,000 in 2060.

### Population

The current immigration level will cause a slight increase in Germany’s population over the next few years. Subsequently, the overall demographic trend will continue. According to the forecast, Germany’s current population is expected to decline from approx. 82 million persons to about 75 million people in 2060. The 66% confidence interval of the forecast shows that the population size will be between about 68 and 81 million in 2060.

We can compare our result with up-to-date projections from the German FSO and from Eurostat. The FSO (2017) provides a couple of scenarios. The total population is projected to be between 60 and 86 million people in 2060, depending on the set of assumptions. Eurostat presents a similar range of variants, resulting in a total population that decreases by 2060 to 63 million in case of no migration, and 87 million if higher migration is assumed (Eurostat 2017).

These scenarios from official agencies are influenced by recent population trends. For the sake of simplicity, we only refer to FSO in the following. The FSO calculated an updated variant, starting with 750,000 net migration in 2016 that declines until 2020. In the long run, 200,000 net immigration was assumed. All in all, this gives an average of 247,000 net migration p.a. In addition, fertility was raised to 1.5 children per woman. This update produces a total population of 76.5 million, a plus of 3.4 million compared with the former variant (with a starting value of 450,000 migration and 1.4 TFR). The main difference between FSO and our forecast is not only the overall development, but also the way in which we get it. As the FSO does not distinguish fertility by nationality, e.g., their TFRs will probably be affected by migration flows.

In addition to the decline in absolute numbers, the German population will also grow older. We expect the median age of 44.5 for men and 46.8 for women in 2013 to continue to rise, peaking in 2050 at 47.2 and 50.1 for men and women, respectively. When the baby boom generation reaches old age, with high mortality, the median age will decline. In 2060, 50% of all women will be at least 49.4 years old and 50% of all men at least 47 years old.

**Fig. 3:**
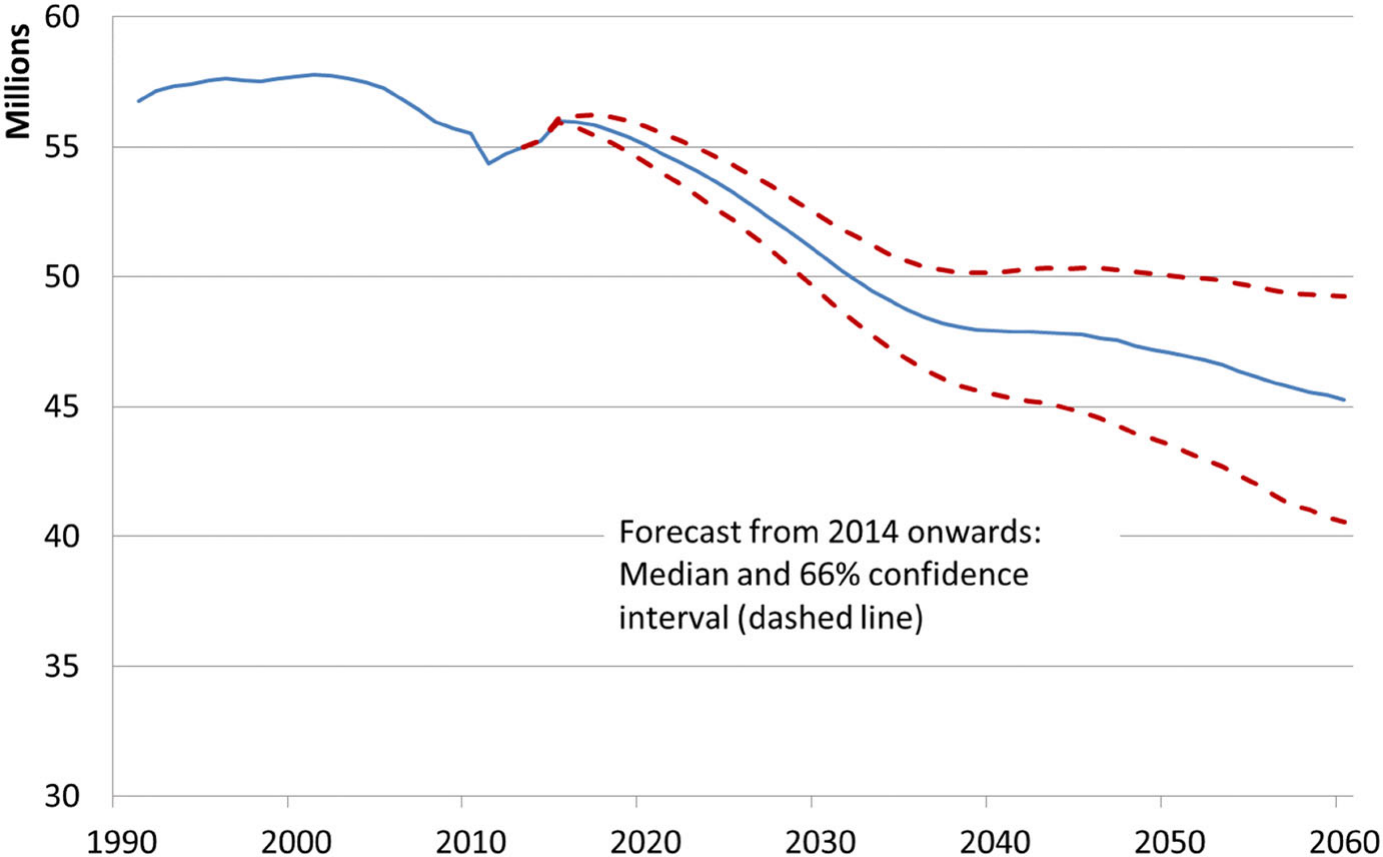
Working-age population (aged 15–66), Germany, 1991–2060, median forecast and confidence interval from 2014 onward. *Source* Federal Statistical Office and own estimations

The working-age population, aged 15–66 years, will experience an above-average decrease. Figure 3 shows an expected decline from approx. 55 million to about 45.3 million. In 2060, it will be almost 18% lower than that in 2013. During the same period, the total population will decline by only 7%. The FSO predicts a working-age population of almost 45 million for 2060, i.e., very close to our forecast. Looking at the confidence bands, however, we recognize some degree of uncertainty, as it ranges from 40.9 to 49.3 million persons.

Once the baby boom generation has reached retirement age, around 2030, the downward trend will slow down noticeably. Around 2050, the decline of the working-age population is predicted to accelerate again. This effect can be explained by a long-run implication of the baby boom cohorts from the 1960s. Considering the mean-age of motherhood in Germany, which was about 28 years of age in 1991, the children of these baby boom cohorts will reach an age beyond the legal retirement age from about 2050 onward (which will be 67 years by then). As this development will not affect the trend of the overall population, the share of the working-age population will decrease more rapidly after 2050.

As long as the birth rate remains well below the level required to maintain the population in the long term, an end to this negative trend is not foreseeable. As expected, the confidence interval widens in the long run. However, even the upper bound in Fig. 3 shows a downward tendency.

### Labor Force Participation

**Fig. 4:**
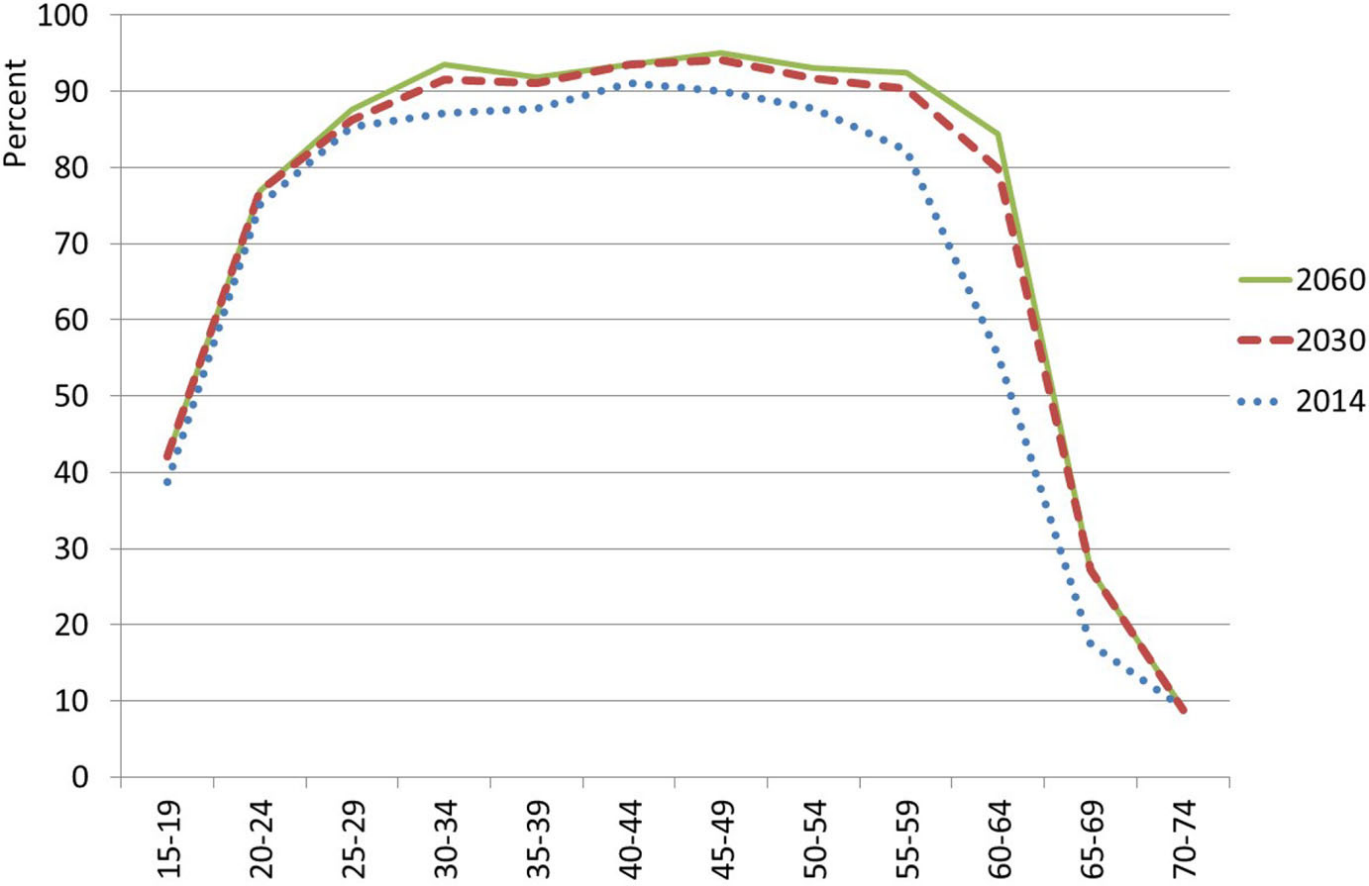
Age-specific labor force participation rates of German women, over selected years. *Sources* Labor Force Survey, own estimations

In the case of German women, a considerable rise in labor force participation is forecast (Fig. 4). In 2014, the labor force participation of women aged 40–49 stood at about 90%. A similarly high participation rate is expected for the group aged 30–59 in 2030. The labor force participation in these age groups will continue to rise until 2060, though to a significantly lesser extent. This development extrapolates what has been observed in Germany for decades and is supported by changes in many socio-economic determinants (part-time work, participation in education, fertility, discouragement of early retirement).

The most substantial increases until 2030 are expected for those aged 55 and older as a result of changes in the old-age pension schemes. As early retirement pathways has been closed step by step since the mid-1990s, we can observe an increasing participation of the elderly for a couple of years. Insofar as the average retirement age has been rising for many years, we should therefore expect a further increase, as the statutory retirement age is set to rise between 2011 and 2030 (but will remain constant at age 67 from 2030 onward).

Among German men, in contrast, there is hardly any potential to increase participation. The LFPR of middle-aged men is very high at 98%, and is expected to remain this level in the future. As with women, higher LFPR among older men can be attributed to the rise in the retirement age. Between 2014 and 2060, labor force participation of people aged 60–64 is expected to rise from 72 to 88%; among those aged 65–69 the LFPR will increase by 12% points to 38%.

The potential LFPR for middle-aged male foreign nationals in 2014 is about 90%. On average, the forecasts until 2060 yield an additional moderate increase of 2–3% points. For 20–24 year olds, who exhibited a labor force participation of about 80% in 2014, a growth of almost 8 percentage points is forecast by 2060. This might be due to spending less time in the education system compared to the German population.

Female foreign nationals participate in the labor market far less than German women. In 2014, only 70% of the female non-Germans aged 35–59 were part of the potential labor force, compared to about 85% of the German women of this age. Although the largest increases in all four demographic groups (men/women, Germans/non-Germans) by 2060 are forecast for female foreign nationals, their labor market participation will remain comparatively low.

**Fig. 5:**
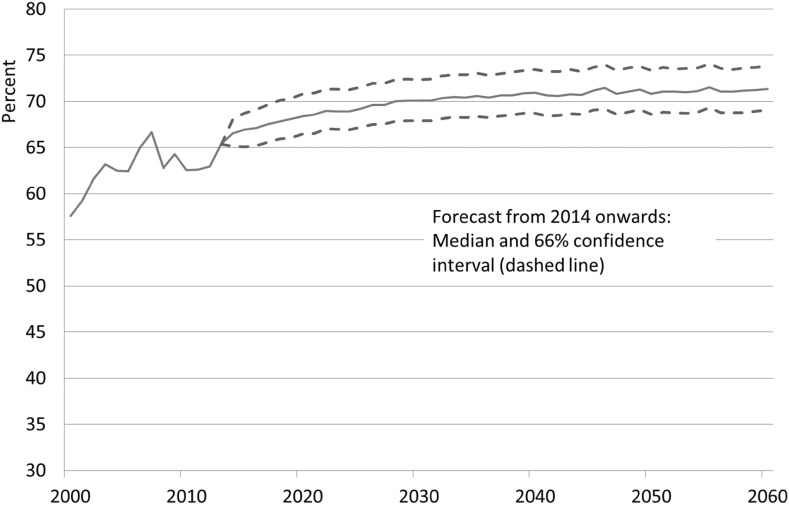
Labor force participation rates of female foreign nationals aged 30–34, 2000–2060, median forecast and confidence interval from 2014 onward. *Sources* Labor Force Survey, own estimations

The example in Fig. 5 shows an increase in the potential LFPR of about 5% points for female foreign nationals aged 30–34. The confidence interval widens in the forecast period from 4.31 (difference in percentage points) in 2020 to 4.46 in 2030 (see Fig. 5). As for the years thereafter, the difference between the upper and the lower bands only slightly increases from 4.73 in 2040 to 4.79 in 2060. This shows that it is possible to derive rather narrow confidence interval using PCA. Also measured by the confidence interval, a stronger labor force participation among this group in the future can be assumed to be very likely.

### Potential Labor Force

**Fig. 6:**
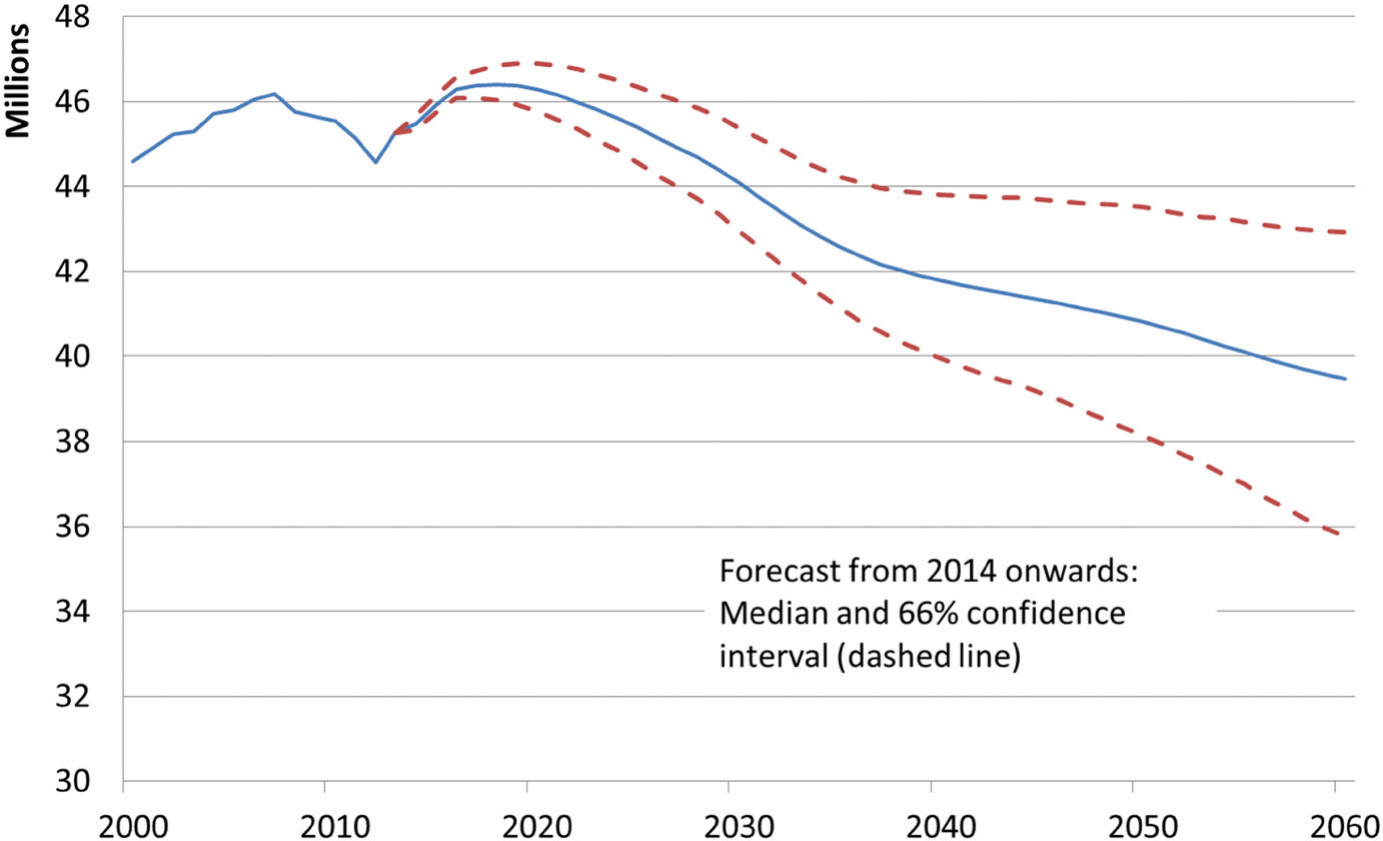
Potential labor force, Germany, 2000–2060, median forecast and confidence interval from 2014 onward. *Sources* Own estimations

According to our forecast the potential labor force will decrease by about 6 million from 45.5 million (2014) to 39.5 million persons by 2060 (Fig. 6). In other words, labor supply can be expected to decline by 13% between 2014 and 2060 while the underlying population of 15–74 year olds is forecast to fall by about 15% and the number of people aged 15–66 by as much as 18%. This underlines the demographic influence on labor supply. The number of baby boom cohorts retiring from working life will be difficult to offset by immigration or higher LFPR.

Once the baby boom generation has retired around 2030, the demographic effect will weaken, as will the decline in the potential labor force. Yet, this development will continue as a result of shrinking cohort sizes. While the confidence interval of the forecast shows considerable uncertainty with respect to the future development of the potential labor force, a constant trend would clearly fall outside the confidence interval.

Similar to the working-age population, the projected downward trend of the potential labor force will accelerate again, starting around 2050. But this development is less pronounced for the labor force than for the working-age population.

The age structure of the potential labor force is largely determined by demographic effects. The share of 15–29 year olds in the potential labor force is about 21% in 2014 and stabilizes at about 20% in the long run (see Table 8). The development of the share of people aged 30–49 primarily reflects the strong rise in the number of births until the mid-1960s (baby boomer) and the drastic drop in the birth rate in the 1970s. Their share has already been in decline since 2000 and is expected to fall further from 53 to 44% by 2060.

**Table 8:** Selected indicators for the German workforce, 2014, 2030, 2050, and 2060. *Source* Own calculations

	2014	2030	2050	2060
in 1000s				
Population, aged 15–74	61,837	59,872	53,987	52,828
Potential labor force	45,478	44,089	40,815	39,461
of which (in % of the labor force)				
Aged 15–29	21	19	20	20
Aged 30–49	44	44	43	44
Aged 50–64	32	31	32	31
Aged 65–74	3	6	5	5
German	96	87	84	82
Foreign	10	13	16	18

At the same time, the number of people aged 50–64 is growing. By 2020, their share of the potential labor force will have grown from 31 to 35% and will decline once the baby boom cohorts start to retire. In the long term, due to our forecast, it will plateau at about 31%. With baby boomers entering the age group 65–74 years and the retirement age simultaneously increasing to 67 years, the share of this group of older workers will increase to 6% by 2030. By 2060, it will decline minimally and stabilize at 5%.

The predicted net emigration will result in a rising number and share of the non-German population. The share of foreigners will almost double between 2014 and 2060, from 10 to 18% (Table 8). This suggests a rising importance of non-German workers for the German labor market.

## Conclusion and Discussion

A stochastic model was developed to forecast population and labor supply in Germany until 2060. Labor supply was quantified using the potential labor force, which in addition to the employed and the unemployed persons also includes the hidden labor force. Calculations were disaggregated in detail, namely by gender and age, and were separated into Germans and non-Germans.

The forecasts of birth rates, mortality rates, naturalizations as well as immigration and emigration are based on the PC method. To forecast the estimated PC, we applied ARMA models. Similarly, the variability of the estimates of the potential LFPR was used for stochastic forecasts and simulations. The stochastic forecast relies on bootstrapping that makes it possible to define forecast intervals with probabilities.

The stochastic model points toward the general demographic trends: the population and the labor force in Germany will decline in the long term. Even the currently extremely high immigration level will not be able to stop this development.

According to the stochastic forecast, the TFR for German women will rise to almost 1.5 children per woman by 2060, with a 66% confidence interval of 1.45–1.6. This growth corresponds to expectations based on the tempo effect. We assume that forecasting with PC covers the tempo effect, simply as it incorporates the cross- and autocorrelation relations between age-specific birth rates (see Vanella 2016). The predicted age-specific birth rates, mentioned above, support this explanation. And looking at the cohorts, our results affirms this shift of motherhood to higher ages, too. Younger cohorts are forecast to have smaller birth rates than older cohorts when they are still young. Later in life, their predicted birth rates are higher than those of older cohorts. Our hypothesis, however, lacks a deeper analysis of the underlying cohort-postponement or of parity-specific fertility rates. We think this would be an interesting and fruitful field for further research, especially when parity-specific fertility data are not available (see Luy and Pötzsch 2010).

Even though we project a rather high and rising level of immigration, net migration effects will be limited. The reason is that emigration also grows, since it depends endogenously on the increasing population of non-Germans. Thus, by 2030, the net migration of foreign nationals will grow to 120,000 persons per year and then remain virtually constant. The confidence interval even includes a negative annual net migration—which Germany last experienced in 1998.

Over the forecast period, the median of all simulations of total population decreases by 7%. The working-age population, aged 15–66, will decrease even more (median − 18%). This is a result of the baby boom generation retiring as well as the small size of subsequent cohorts. Although the group-specific potential LFPR, at least for the middle-age groups and for the elderly, are rather high in international comparison, they will increase even further. However, the population decline outweighs this growth in labor force participation. The stochastic forecast for the potential labor force therefore yields an average decline by 6 million workers, from about 46 million at present to under 40 million in 2060. For reasons of comparison, the current employment statistic in Germany counts about 43.6 million workers. The forecast shows, furthermore, a rising importance of foreign nationals for the German labor market.

Our method foresees a clear reduction of the potential labor force, even if stochastic variation is taken into account. While a lower labor force per se is not critical for an economy, frictions in the adjustment process can lead to socially and economically harmful impacts (Weber 2016). By the same token, the financing of the social security systems becomes increasingly problematic. Therefore, our result emphasizes the need for both active labor market policies to promote domestic manpower reserves, which also includes a rise in working hours, as well as a labor market-oriented long-term immigration policy—as unnecessary as this may seem in the current situation with net migration amounting to over 1 million in 2015.

Of course, our stochastic approach has some limitations. For example, we could query the precision of the estimated variances that came into effect in the bootstrapping method. Some of our times series are relatively short, but as a tool for estimating the variances (and auto-/covariances in case of the PC), the length of the time series are in line with other studies. In general, it is important that the time series cover different historical conditions and will still be relevant for the forecast horizon (for a discussion of the pros and cons of very long historical data, see Alders et al. 2007).

A typical limitation concerns the modeling of migration, which is subject to considerable uncertainty. Therefore, further improvements of our model address the migration component, by expanding the immigration flows by country or region of origin. Another refinement aims at labor participation, where PCA could be applied in future as well.

## Appendix

See Tables 9, 10, 11 and 12.

**Table 9:** Principal components analysis for immigration of German women. *Source* Own calculations

	Eigenvalues	Difference	Proportion	Cum. Eigenvalue
1	48.8881	30.0220	0.5372	48.8881
2	18.8661	10.5280	0.2073	67.7542
3	8.3381	3.1676	0.0916	76.0923
4	5.1705	3.2430	0.0568	81.2628
5	1.9274	0.2093	0.0212	83.1902
6	1.7181	0.5785	0.0189	84.9084
7	1.1396	0.3786	0.0125	86.0480
8	0.7610	0.0434	0.0084	86.8090
9	0.7176	0.1479	0.0079	87.5266
10	0.5697	0.1076	0.0063	88.0964
11	0.4622	0.1130	0.0051	88.5585
12	0.3492	0.0233	0.0038	88.9077

Log (share of immigrants aged i in the total immigration), for all ages

Sample 1991–2014 (24 observations)

**Table 10:** Principal components analysis for the emigration of German women. *Source* Own calculations

	Eigenvalues	Difference	Proportion	Cum. Eigenvalue
1	54.5810	38.1152	0.5998	54.5810
2	16.4658	10.6819	0.1809	71.0467
3	5.7839	2.6015	0.0636	76.8306
4	3.1824	1.4754	0.0350	80.0129
5	1.7070	0.1312	0.0188	81.7199
6	1.5758	0.5029	0.0173	83.2957
7	1.0729	0.0811	0.0118	84.3686
8	0.9918	0.2070	0.0109	85.3604
9	0.7848	0.0627	0.0086	86.1452
10	0.7221	0.0617	0.0079	86.8673
11	0.6604	0.1094	0.0073	87.5277
12	0.5511	0.0268	0.0061	88.0788

Log (share of emigrants aged i in the total emigration), for all ages

Sample 1991–2014 (24 observations)

**Table 11:** Principal components analysis for the fertility rates of German women. *Source* Own calculations

	Eigenvalues	Difference	Proportion	Cum. Eigenvalue
1	28.6568	25.0779	0.8188	28.6568
2	3.5789	2.1985	0.1023	32.2358
3	1.3804	0.7834	0.0394	33.6162
4	0.5970	0.3349	0.0171	34.2132
5	0.2622	0.0992	0.0075	34.4754
6	0.1630	0.0210	0.0047	34.6384
7	0.1419	0.0690	0.0041	34.7803
8	0.0729	0.0380	0.0021	34.8533
9	0.0350	0.0040	0.0010	34.8882
10	0.0310	0.0062	0.0009	34.9192
11	0.0248	0.0047	0.0007	34.9440
12	0.0201	0.0092	0.0006	34.9641

Log (Fertility rate)

Sample: 1991–2014 (24 observations)

**Table 12:** Correlogram/Q-statistic for the 2nd PC of the birth rates of German women (see equation from Table 3). *Source* Own calculations

	Auto correlation	Partial correlation	Q-statistic	Probability
1	− 0.175	− 0.175	0.737	
2	0.133	0.106	1.187	
3	0.048	0.091	1.248	0.264
4	0.110	0.123	1.589	0.452
5	0.027	0.052	1.612	0.657
6	0.056	0.039	1.712	0.789
7	− 0.200	− 0.226	3.099	0.685
8	0.135	0.034	3.775	0.707
9	− 0.246	− 0.210	6.207	0.516
10	− 0.052	− 0.137	6.326	0.611
11	0.066	0.132	6.540	0.685
12	− 0.123	− 0.038	7.356	0.691

Probabilities for the first two lags are not valid, as an AR(2) was estimated

## Footnotes

1 Statistics New Zealand publishes a stochastic forecast, but we have not found any publication; see http://m.stats.govt.nz/tools_and_services/nzdotstat/tables-by-subject/population-projections-tables.aspx#NLFP.

2 The potential labor force covers the age interval from 15–74 years. For statistical reasons, it was assumed that the LFPRs of the oldest group (65 +) will remain largely unchanged in future. In addition, due to the retirement age, hidden unemployment should be important only up to the age of 64.
